# Willingness to Get a COVID-19 Vaccine and Its Potential Predictors in Costa Rica: A Cross-Sectional Study

**DOI:** 10.7759/cureus.18798

**Published:** 2021-10-15

**Authors:** Carlos A Faerron Guzmán, Pablo Montero-Zamora, Carolina Bolaños-Palmieri, Juliana Araya-Amador, Jorge Benavides-Rawson, María L Ávila-Agüero

**Affiliations:** 1 Medicine, University of Maryland, Baltimore, USA; 2 Public Health Sciences, Miller School of Medicine, University of Miami, Miami, USA; 3 Global Health, Inter American Center for Global Health, San José, CRI; 4 Physiology, Escuela Autónoma de Ciencias Médicas de Centro América (Universidad de Ciencias Médicas), San José, CRI; 5 Anthropology, George Washington University, Washington DC, USA; 6 Pediatric Infectious Diseases, Hospital Nacional De Niños "Dr. Carlos Sáenz Herrera", San José, CRI; 7 Pediatric Infectious Diseases, Center for Infectious Disease Modeling and Analysis, Yale School of Public Health, New Haven, USA

**Keywords:** costa rica, immunization, attitudes, covid-19, hesitancy, pandemic

## Abstract

Since 2020, the COVID-19 pandemic has had a significant impact on morbidity and mortality around the world. As one of the most successful and cost-effective health interventions for preventing infectious diseases, immunization against SARS-CoV-2, is at the moment the most effective strategy for controlling the current pandemic. Despite the high vaccine acceptance rates that countries such as Costa Rica have shown in the past, the public acceptance of the COVID-19 vaccine is still uncertain. The purpose of this study was to examine adults’ willingness to get a novel COVID-19 vaccine and its potential predictors in Costa Rica. We conducted a cross-sectional study comprising a sample of 4717 adult participants living in Costa Rica who participated in a voluntary electronic survey regarding their intent to get a future COVID-19 vaccine. Results suggest that a high percentage (86.1%) of Costa Ricans aged 18 or more would be willing to be vaccinated once a safe and effective vaccine is approved and distributed in the country. Some relevant significant predictors of willingness to get a COVID-19 vaccine in Costa Rica were higher income, being male, work in the health care sector, and having a relative or a close acquaintance diagnosed with COVID-19. Our findings suggest that it is essential to concentrate efforts on the immediate development of culturally appropriate communication, dissemination, and implementation strategies to maximize immunization against SARS-CoV-2 in Costa Rica.

## Introduction

The COVID-19 pandemic has brought a significant burden of morbidity and mortality worldwide, causing the loss of nearly 20.5 million years of life by early 2021 [1]. Specifically, Costa Rica identified the first case of COVID-19 on March 6, 2020. By the beginning of December 2020, when this study took place, the country reported almost 150,000 cases and 1,850 deaths, and its population was still waiting for the arrival of the first type of COVID-19 vaccine approved by the country’s Ministry of Health and the U.S. Food and Drug Administration (i.e., Pfizer-BioNTech) [2, 3]. 

Immunizations are one of the most successful and cost-effective health interventions for preventing infectious diseases, and vaccines against SARS-CoV-2 are, at the moment, the most effective strategies for controlling the current pandemic [4]. Despite this, there is uncertainty about the public acceptance of the COVID-19 vaccine [5, 6]. The reasons people refuse vaccines are complex. Even before the pandemic, low willingness to get vaccines (i.e., vaccine hesitancy) has been a public health challenge, and recently, the WHO declared it one of the ten most significant issues to address [7, 8]. For instance, a recent multi-center study [9] assessed the willingness to get the COVID-19 vaccine in 19 countries, identified that acceptance rates for vaccination considerably differed in each location. In this study, 71.5% of participants reported they would be very or somewhat likely to take a COVID-19 vaccine [9]. In addition, Paul, et al. report that distrustful attitudes towards vaccination were higher amongst individuals from ethnic minority backgrounds, with lower levels of education, lower annual income, poor knowledge of COVID-19, and poor compliance with government COVID-19 guidelines [10]. 

Low willingness to get a vaccine could represent a major problem in the global efforts to control the current COVID-19 pandemic [11]. In countries such as Costa Rica, vaccination programs are recognized to be solid, modern for all populations including adults [12, 13]. However, the SARS-CoV2 pandemic represents a new challenge for the nation, by having to vaccinate a large number of adults who are not used to getting vaccines in a very short time period. That is why it is necessary to know the concerns, doubts, and perceptions of this population regarding vaccines and their intention to get them or not. In response to the needs expressed by local health authorities, our team conducted a cross-sectional study to examine adults’ willingness to get a future COVID-19 vaccine and its potential predictors in Costa Rica. Our findings have fundamental implications for guiding health authorities’ communication strategies as COVID-19 vaccines become more widely available.

## Materials and methods

Design, participant’s selection, and data collection

A cross-sectional study was carried out through a survey about the possible acceptance of a vaccine against COVID-19 in Costa Rica with 4717 participants (aged 18 or more). According to the most recent estimations from the National Institute of Statistics, by 2020, Costa Rica had approximately 5 million inhabitants, from whom 29% were 18-34 years, 26% were 35-54 years, and 19% had more than 54 years [14]. We used a non-probabilistic, snowball convenience sampling method to roll out the survey [15]. The surveys were distributed without limitations by the research team members and research subjects through different social media platforms (e.g., Facebook and Twitter). Due to the high penetrance of online messaging services in the region, we also distributed the survey through messaging services (i.e., WhatsApp). The survey was open from Wednesday, December 2 through Sunday, December 6, 2020. All study procedures were approved by the Institutional Scientific Ethics Committee of the Costa Rican National Children's Hospital.

Measures

Participants’ willingness to get a COVID-19 vaccine was measured by asking: “When a safe and effective vaccine against COVID-19 is available in Costa Rica, would you be willing to get vaccinated?” Research subjects were also asked to optionally register a short explanation of their answer. Responses were recorded using a five-point Likert scale (e.g., completely disagree = 0, completely agree = 4). We additionally collected participants’ sociodemographic data. Age was coded in categories from 18-34, 35-54, and 55 or more years. Income was classified using the following categories: less than 200,000 colones, between 450,000 and 750,000 colones, between 750,000 and 1,150,000 colones, and more than 1,150,000 colones (1 dollar = 624 colones). Education level was classified as complete or incomplete school, complete or incomplete high school, complete or incomplete technical career, complete or incomplete college. Sex was defined as male, female or other. We also collected information on whether the person taking the survey worked in the health care sector and whether they had a family member with a COVID-19 confirmed diagnosis within the last six months.

Data analysis

Data analysis was performed using the statistical package STATA 16 (StataCorp, College Station, USA) [16]. Multiple logistic regression modeling [17] was used to evaluate the association between sociodemographic predictors (i.e., independent variables) and participants’ willingness to get a COVID-19 vaccine (i.e., dependent variable). The dependent variable was coded as 1 when the respondent answered, “strongly agree” or “agree” and was coded as 0 for any other response. Likewise, we evaluated all the interactions between sex and the rest of the independent variables to detect possible sex differences. We kept into the final model only those interactions that showed to be statistically significant (i.e., p <0.05). Under these considerations, to evaluate the potential predictors of willingness to get a COVID-19 vaccine in Costa Rica, the following multiple logistic regression model was adjusted and interpreted:

log [pi / (1 - pi)] = βO + β1 AGE + β2SEX + β3 INCOME + β4EDUCATION + β5OCUPATION + β6HEALTH_OCCUPATION + β7NATIONALITY + β8PROVINCE + β9ACQUAINTANCES_COVID-19 + β10INCOME*SEX + β11OCUPATION*SEX- + β12HEALTH_OCCUPATION*SEX

Where pi is equal to the probability of willingness to get a COVID-19 vaccine of the i-th person, HEALTH_OCCUPATION is the dichotomous variable referring to work in the health sector, and ACQUAINTANCES_COVID-19 is the dichotomous variable referring to having a relative or a close person diagnosed with COVID-19. Finally, HEALTH_OCCUPATION*SEX corresponds to the dichotomous variable referring to working in the health sector and its respective interaction with sex. The final model used for interpretation kept all previously mentioned predictors.

## Results

Results suggest that a high percentage (86.1%) of the Costa Rican population would be willing to get a novel COVID-19 vaccine when a safe and effective vaccine became approved and available. As shown in Table 1, of the total amount of people that took the survey, 42% were between the ages of 18 and 34, 43% were between the ages of 35 and 54, and 15% were 55 or older at the time of the survey. Overall, no significant associations were found between the categories of age, education, and nationality in general. Additional analyses were conducted using different reference education categories; however, no changes in results were found. Little geographic heterogeneity was observed in the respondents' willingness to get the vaccine, resulting in a general non-significant difference in vaccine willingness among territorial divisions in the country.

**Table 1:** Results of multiple logistic regression showing predictors of willingness to get a COVID-19 vaccine AOR= Adjusted Odds Ratio. SE = Standard Error. 95% CI = 95% Confidence Interval. Ref = reference ^t^p < .10. *p < .05. **p < . 01. **p < .001.

Independent variable	n (%)	AOR	SE	95% CI		p-value
Age, years						
<34 years old)	1976 (42%)	Ref				
34 - 55 years	2057 (43%)	1.09	.115	.890	1.34	.395
55 years or older	696 (15%)	1.10	.182	.801	1.52	.539
Sex						
Female	3013 (64%)	Ref				
Male	1692 (36%)	2.12	.632	1.18	3.80	.011^**^
Income						
>1,150,000 colones	2212 (47%)	Ref				
750,000 - 1,150,000 colones	973 (20%)	0.86	.118	.658	1.12	.273
450,000 - 750,000 colones	701 (15%)	0.69	.104	.510	.923	.013^**^
<200,000 colones	843 (18%)	0.72	.117	.522	.987	.041^*^
Education						
Technical career	252 (5%)	Ref				
Complete or incomplete school	380 (8%)	.872	.121	.665	1.14	.323
Complete or incomplete high school	51 (1%)	.690	.104	.513	.928	.014^*^
Complete or incomplete college	4046 (86%)	.721	.118	.523	.995	.046^*^
Occupation						
Self-employed	559 (12%)	Ref				
Currently unemployed	206 (4%)	2.79	.887	1.50	5.20	.001^**^
State employee	736 (16%)	1.46	.268	1.02	2.09	.038^*^
Private company employee	1519 (32%)	1.53	.257	1.10	2.13	.010^*^
Employee autonomous state institution	376 (8%)	1.43	.340	.903	2.28	.126
Student	611 (13%)	.545	.182	.282	1.05	.070^t^
Employer - active partner	100 (2%)	2.62	.593	1.68	4.09	.000^***^
Retired	273 (6%)	1.77	.519	.996	3.14	.052
Domestic service	59 (1%)	1.35	.528	.630	2.91	.438
Other	290 (6%)	.993	.222	.641	1.54	.977
Health sector worker						
No	1024 (22%)	Ref				
Yes	3630 (78%)	.986	.126	.768	1.26	.913
Nationality						
Foreign	93 (2%)	Ref				
Costa Rican	4638 (98%)	1.26	.384	.695	2.28	.446
Province						
San Jose	2207 (47%)	Ref				
Alajuela	678 (14%)	.894	.115	.695	1.15	.383
Cartago	624 (13%)	1.05	.147	.800	1.38	.715
Guanacaste	156 (3%)	1.10	.287	.664	1.84	.702
Heredia	803 (17%)	1.23	.161	.955	1.59	.109
Limon	79 (2%)	.942	.324	.480	1.85	.863
Puntarenas	174 (4%)	1.24	.314	.755	2.04	.395
Family or friend with COVID-19						
No	1073 (23%)	Ref				
Yes	3630 (77%)	1.30	.132	1.06	1.58	.010^*^
Income X Sex						
750,000 - 1,150,000 colones X Male	298 (6%)	1.12	.297	.668	1.88	.662
450,000 - 750,000 colones X Male	236 (5%)	2.79	.964	1.41	5.49	.003
<200,000 colones X Male	241 (5%)	1.50	.455	.827	2.71	.182
Occupation X Sex						
Currently unemployed X Male	57 (1%)	.219	.122	.073	.654	.007^**^
State employee X Male	57 (1%)	.813	.309	.386	1.71	.586
Private company employee X Male	211 (5%)	1.02	.458	.428	2.46	.953
Employee autonomous state institution X Male	206 (4%)	.740	.234	.398	1.37	.341
Student X Male	154 (3%)	1.39	.836	.426	4.52	.587
Employer - active partner X Male	661 (14%)	.506	.215	.219	1.16	.109
Retired X Male	273 (6%)	.581	.284	.223	1.51	.266
Domestic service X Male	0 (0%)	1.00				
Health sector worker X Sex						
Yes X Male	344 (7%)	.572	.134	.362	.904	.017^**^

In general, compared with women, men reported a greater likelihood to get vaccinated against COVID-19 (adjusted odds ratio [AOR] = 2.12, p = .011). Furthermore, compared to those whose income was more than 1,150,000 colones (approx. $1800), participants who reported an income between 500,000 colones (approx. $700) and 750,000 colones (approx. $1200) showed 31% less likelihood of agreeing to be vaccinated against COVID-19 (AOR = .690, p = .014). Similarly, those who reported an income lower than 200,000 colones (approx. $300) showed Also, those who did not report having a relative or close friend diagnosed with COVID-19 had a 30% greater chance of agreeing to get the vaccine (AOR = .721, p = .046). 

Among the different occupations, we found that compared with participants who reported being self-employed - by the time of our survey - significantly greater chances of accepting vaccinations were observed in those who (1) reported being unemployed (AOR = 2.79, p = .001), (2) working for the state (AOR = 1.46, p = .038), (3) working in a private company (AOR = 1.53, p = .010), and studying (AOR = .545, p = .070). Also, we observed that unemployed men reported almost 78% fewer odds of accepting to be vaccinated (AOR = .219, p = .007). Finally, our findings suggest that men working in the health sector are 43% less likely to agree to be vaccinated against COVID-19 compared to men who are not part of the health sector (AOR =.572, p = .017). 

## Discussion

This is the first study of which we are aware that reports willingness to get a COVID-19 vaccine in Costa Rica. Our results suggest that Costa Rica has a generally high acceptance rate to the use of COVID-19 vaccines. Predictors such as higher income, being male, working in the health care sector, being unemployed, working for the state, working in a private company, studying, and having a relative or close person diagnosed with COVID-19, were significant predictors of willingness to get a COVID-19 vaccine. We did not find any association between vaccine willingness and age, education, or nationality in general. Important moderation effects within males were found suggesting a need for additional efforts to communicate vaccination benefits to this population especially those working in the State and health sectors.

Our results suggest that the high willingness to get a COVID-19 vaccine among Costa Ricans should not come as a surprise, as prior evidence found similar findings reported in low- middle-income countries (LMICs) such as Costa Rica [11, 18]. Particularly, Costa Rica has been reporting a long tradition of high levels of acceptance towards vaccination, and it has a robust National Immunization Program that reaches over 90% of the population [12]. The “Report on the results of the study of socio-political opinion” of the School of Political Sciences of the University of Costa Rica in April 2019 [19], shows that when faced with the question of whether there are Costa Ricans opposed to vaccines, a large majority of citizens (96 %) approve of vaccinating everyone when appropriate, while only 4% disapprove. The aforementioned is pre-pandemic data, and unfortunately during the pandemic, false news and the heightened uncertainty could have negatively affected the confidence in vaccines [19, 20]. However, our analysis’s preliminary results suggest that within the group less willing to get the vaccine, misinformation and disinformation about the vaccine’s benefits might influence people’s perception of the vaccine's safety. Even though it is beyond the scope of this study, further research on the causes of misinformation and disinformation could prove vital to counter their effects. 

In the open responses from the group less willing to get the vaccine, the most salient themes include safety concerns, mistrust in health authorities, and lack of trust or suspicion about pharmaceutical companies.

Our findings suggest that higher income, being male, and having a relative or a close person diagnosed with COVID-19 are important predictors for having greater acceptance rates. The finding that higher income predicts a greater vaccine acceptance in Costa Rica, aligns with another study conducted in LMICs [21]. Also, our results are consistent with those reported by Lindholt et al. (2019), in which being male was associated with higher vaccine acceptance [22]. Based on our general findings, we suggest that Costa Rican health authorities must create communication and education strategies on vaccines that consider the population's sex, income, occupation, health literacy levels, and prevailing attitudes toward health authorities and pharmaceutical companies. Strategies to promote vaccine acceptance must directly address the specific concerns or misconceptions of Costa Ricans and counter sources of mistrust. In order to achieve cultural resonance, these efforts must incorporate organized civil society, religious organizations, community leaders, and the private sector.

Clear and consistent communication by health authorities and allies will be essential to build public confidence regarding the COVID-19 vaccine. This communication should include elements such as: the level of the effectiveness of the different vaccines, the time required for the protection and generation of antibodies, the vaccination schedule for the different vaccine types, how the different vaccines work, vaccine safety and efficacy reports, vaccine development process, vaccine approval process and the importance of herd immunity.

This study had some limitations that are worth noting. As a cross-sectional study, it only analyzes the situation at a particular moment in time. Additionally, we did not use a randomized sample, and the issue of representativeness (or lack of) of the general Costa Rican population must be highlighted. Finally, there is also the possibility that the survey had social desirability bias [23] as it was mainly distributed through channels associated with the health sector.

## Conclusions

The results suggest that a high percentage of the population would be willing to be vaccinated once a safe and effective COVID-19 vaccine is approved and distributed. However, there are still significant barriers to consider improving dissemination in the vaccination process. According to our findings, it is essential to concentrate efforts on the immediate development of culturally appropriate communication, dissemination, and implementation strategies aimed mainly at adult women in general, unemployed, people working in the health sector, and those people who still do not have relatives or close friends who have been diagnosed with COVID-19.
